# Supplementary material for: Dissolved gases from pressure changes in the lungs elicit an immune response in human peripheral blood
Source: Bioeng Transl Med. 2024 Apr 16;9(5):e10657. doi: 10.1002/btm2.10657 (PMC11561805; doi:10.1002/btm2.10657)
Supplement: Supplementary file 1 — Data S1: Supporting Information. [file BTM2-9-e10657-s001.docx]

Supporting Information for:

**Dissolved gases from pressure changes in the lungs elicit an immune response in human peripheral blood**

Abigail G. Harrell,^a^ Stephen R. Thom,^b^ and C. Wyatt Shields IV^a,c,†^

^a^ Department of Chemical and Biological Engineering, University of Colorado Boulder, Boulder, CO 80303, United States

^b^ Department of Emergency Medicine, University of Maryland School of Medicine, Baltimore, MD 21201, United States

^c^ Biomedical Engineering Program, University of Colorado Boulder, Boulder, CO 80303, United States

^†^Corresponding author: Charles.Shields@colorado.edu

Supplementary Materials

Table S1. Antibodies used for flow cytometry.

Supplementary Figures

Figure S1. Confocal microscopy image of human lung-on-a-chip device.

Figure S2. Panel organization for distinguishing and phenotyping monocytes, dendritic cells, and

neutrophils.

Figure S3. Immune cell gating.

Figure S4. Standard curves from multiplexed ELISA.

**I.** **Supplementary Materials**

**Table S1. Antibodies used for flow cytometry.** Extracellular antibodies are indicated with an asterisk (*) and intracellular antibodies are represented with a hashtag (^#^). As a disclaimer, Super Bright antibody conjugates may induce the excitement of the other fluorescent antibodies present within a panel. We gated the resultant spectra to minimize potential crosstalk from the Super Bright antibody conjugates; however, we recommend using a Super Bright staining buffer or avoiding Super Bright antibody conjugates in studies that involve panels comprising multiple fluorescent antibodies.

| **Antibody target** | **Fluorophore** | **Host/isotype** | **Clone** | **Supplier** |
| --- | --- | --- | --- | --- |
| CD11b* | PE-Cyanine7 | Mouse / IgG1, kappa | ICRF44 | Thermo Fisher |
| CD11c* | PE-Cyanine5.5 | Mouse / IgG1, kappa | 3.9 | Thermo Fisher |
| CD80* | Super Bright 436 | Mouse / IgG1, kappa | 2D10.4 | Thermo Fisher |
| HLA DR/DP* | Super Bright 600 | Mouse / IgG2a | HL-38 | Thermo Fisher |
| CD86* | PE | Mouse / IgG2b, kappa | IT2.2 | ThermoFisher |
| CD14* | PE-Cyanine5 | Mouse / IgG1, kappa | 61D3 | Thermo Fisher |
| Arg-1^#^ | eFluor 450 | Rat / IgG2a, kappa | A1exF5 | Thermo Fisher |
| HIF-1𝛼^#^ | PE | Mouse / IgG1, kappa | Mgc3 | Thermo Fisher |
| CD66b* | PE-Cyanine7 | Mouse / IgM, kappa | G10F5 | Thermo Fisher |
| MPO^#^ | PE | Mouse / IgG1 | MPO455-8E6 | Thermo Fisher |
| NE^#^ | Alexa Fluor 405 | Mouse / IgG1 | 950317R | Bio-Techne |
| CD18* | PE | Mouse / IgG1 | MEM-48 | Thermo Fisher |
| CD41a* | Super Bright 436 | Mouse / IgG1, kappa | HIP8 | Thermo Fisher |

**II.** **Supplementary Figures**

**
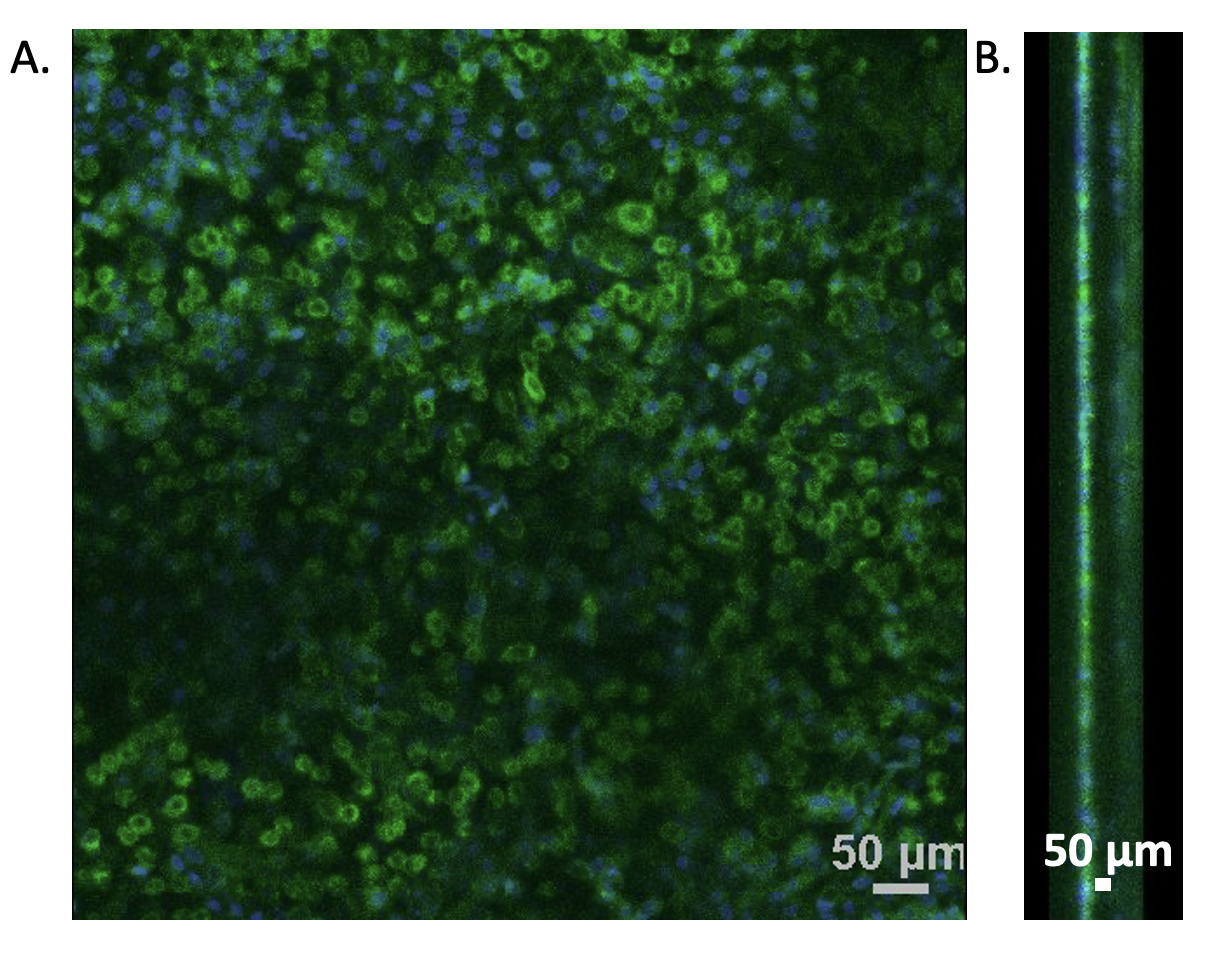
**

**Figure S1. Confocal microscopy image of human lung-on-a-chip device.** (A) Human microvascular endothelial cells (HMVEC-L) cultured in the bottom channel of a human lung-on-a-chip device. (B) Cross-section of the bottom channel cell layer (left), 50 µm membrane, and top channel cell layer (right). Human pulmonary artery endothelial cells (HPAECs) are not shown due to limited resolution through the device thickness. Images are only for illustrative purposes as a high cell passage number was used.

**
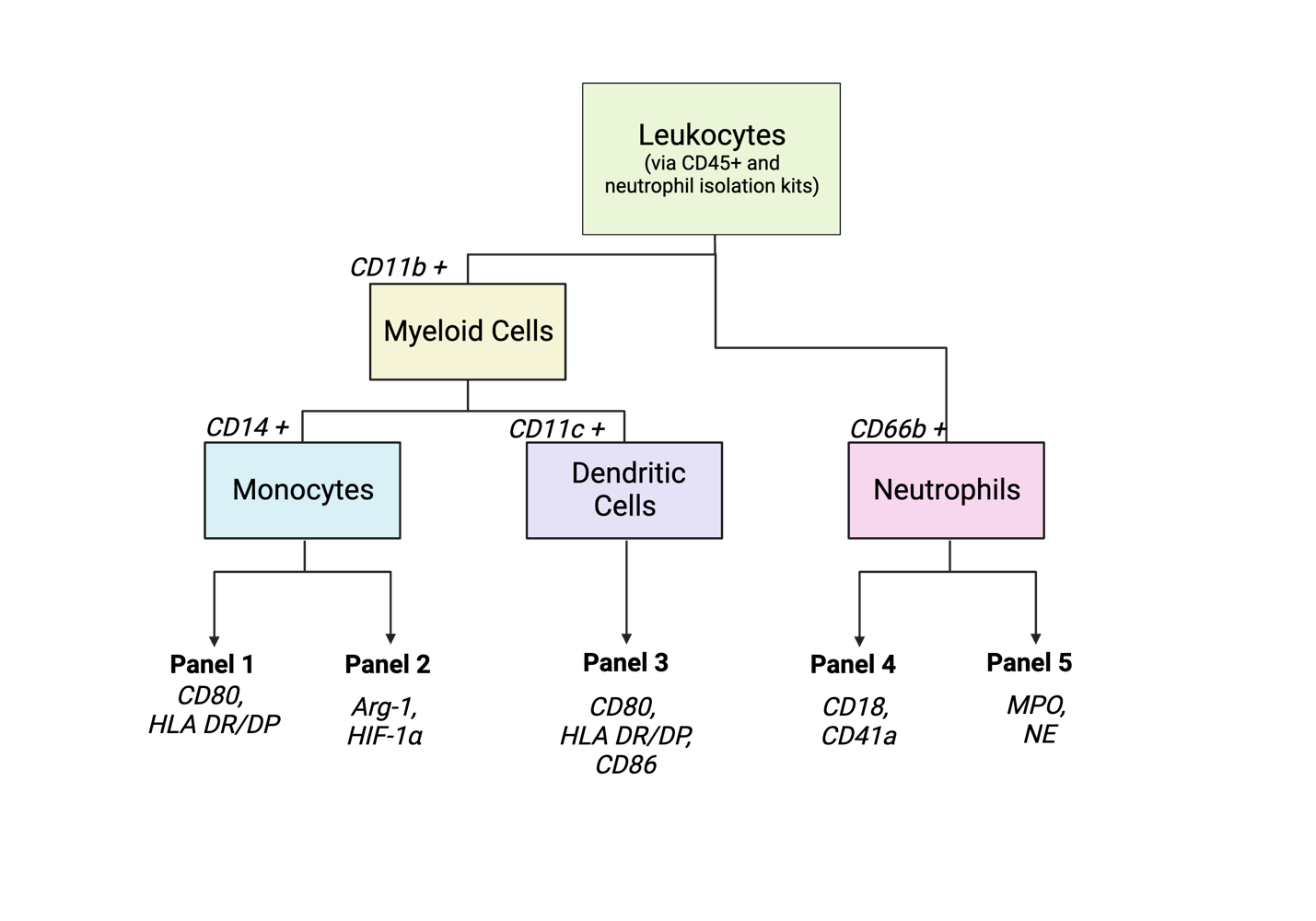
**

**Figure S2. Panel organization for distinguishing and phenotyping monocytes, dendritic cells, and neutrophils.**

**
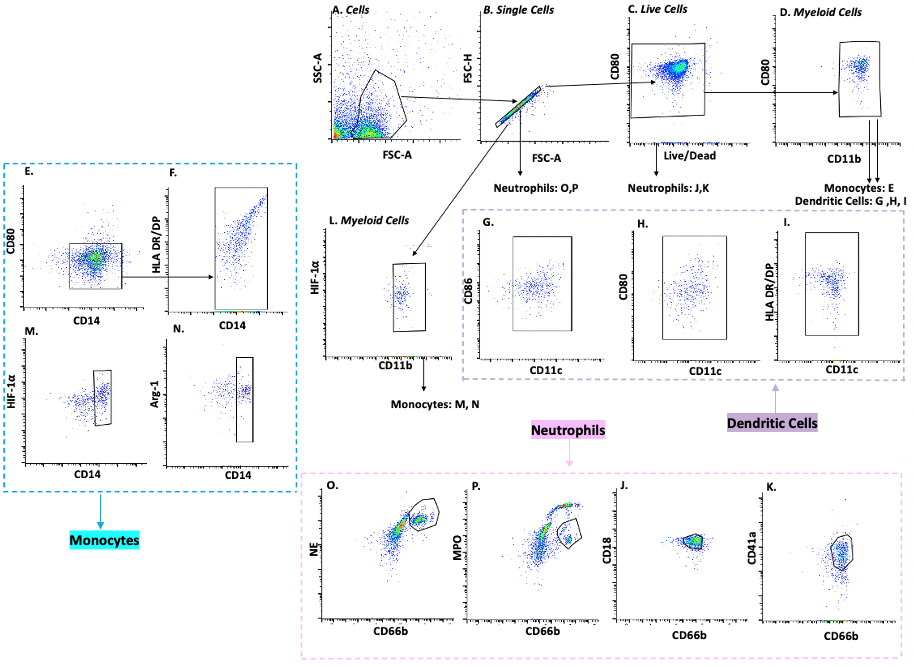
**

**Figure S3. Immune cell gating.** Hierarchical gates were drawn for (A) cells, (B) single cells, (C) live cells, (D) myeloid cells, (E-F) extracellular markers on monocytes, and (G-I) extracellular markers on dendritic cells. Subplots were drawn from (C) to identify (J-K) extracellular markers on neutrophils. To identify intracellular markers in monocytes, which are inherently non-viable due to the fixation / permeabilization protocol, a subplot was drawn from (B) to identify (L) myeloid cells and (M-N) intracellular markers in monocytes. Subplots were drawn from (B) to identify (O-P) intracellular markers in neutrophils. In the *Main Text,* Figure 3 displays the relative fold-change in median fluorescence intensity for (O) NE, (P) MPO, (J) CD18, and (K) CD41a (pink dotted lines). Figure 4 displays the relative change in median fluorescence intensity for (E) HLA DR/DP, (F) CD80, (M) HIF-1𝛼, and (N) Arg-1 (blue dotted lines). Figure 5 displays the relative fold-change in median fluorescence intensity for (G) CD86, (H) CD80, and (I) HLA DR/DP (purple dotted lines).


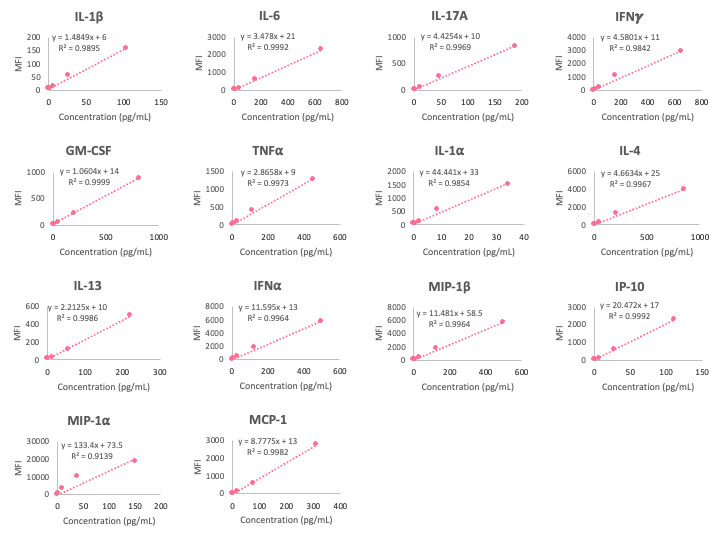


**Figure S4. Standard curves from multiplexed ELISA.** Eight different concentrations were used for each of the fourteen cytokines and chemokines shown in Figure 6 of the *Main Text* to determine standard curves by fitting a linear regression to the data points within the linear regions.
